# Human transgenerational responses to early-life experience: potential impact on development, health and biomedical research

**DOI:** 10.1136/jmedgenet-2014-102577

**Published:** 2014-07-25

**Authors:** Marcus Pembrey, Richard Saffery, Lars Olov Bygren, Network in Epigenetic Epidemiology

**Affiliations:** 1 School of Social & Community Medicine, University of Bristol, Bristol, UK; 2 UCL Institute of Child Health, London, UK; 3 Murdoch Childrens Research Institute, Parkville, Australia; 4 Department of Paediatrics, University of Melbourne, Parkville, Australia; 5 Department of Biosciences and Rehabilitation, Karolinska Institutet, Huddinge, Sweden; 6 Department of Community Medicine and Rehabilitation, Umeå University, Umeå, Sweden

**Keywords:** transgenerational, Epigenetics, miRNAs, epigenetic epidemiology, overkalix

## Abstract

Mammalian experiments provide clear evidence of male line transgenerational effects on health and development from paternal or ancestral early-life exposures such as diet or stress. The few human observational studies to date suggest (male line) transgenerational effects exist that cannot easily be attributed to cultural and/or genetic inheritance. Here we summarise relevant studies, drawing attention to exposure sensitive periods in early life and sex differences in transmission and offspring outcomes. Thus, variation, or changes, in the parental/ancestral environment may influence phenotypic variation for better or worse in the next generation(s), and so contribute to common, non-communicable disease risk including sex differences. We argue that life-course epidemiology should be reframed to include exposures from previous generations, keeping an open mind as to the mechanisms that transmit this information to offspring. Finally, we discuss animal experiments, including the role of epigenetic inheritance and non-coding RNAs, in terms of what lessons can be learnt for designing and interpreting human studies. This review was developed initially as a position paper by the multidisciplinary *Network in Epigenetic Epidemiology* to encourage transgenerational research in human cohorts.

## Background

### The challenge of common chronic disease

The focus of public health is increasingly on chronic disorders, such as diabetes, cardiovascular disease, mental health and cancer manifesting in adulthood. Unlike monogenic (Mendelian) disorders or infectious disease, where the known primary cause permits an aetiological classification and rational approach to management, common diseases are generally multifactorial in origin with diverse genetic and environmental determinants. This contributes to the highly heterogeneous nature of such conditions, which remain an aetiological and management challenge. A multidisciplinary perspective, which includes genetic analysis, is recognised as the most appropriate approach to studying such conditions.^1–3^ Genetic associations are now routinely integrated with long-established epidemiological analysis of nutrition, social and other exposures through which health and disease have been traditionally explored.

### Extending epidemiology beyond genetics and a single generation

For many years, parental and ancestral experience has been regarded as contributing only indirectly across generations. Clearly, such ‘cultural transmission’, which includes parental nurturing behaviour and social patterning of all kinds, does play an important role in shaping the phenotype/traits of progeny. This is an area of active research integrating information on social circumstances, skills, health and mortality, sometimes across three generations of the same lineage.^4^ ^5^

**Figure 1:**
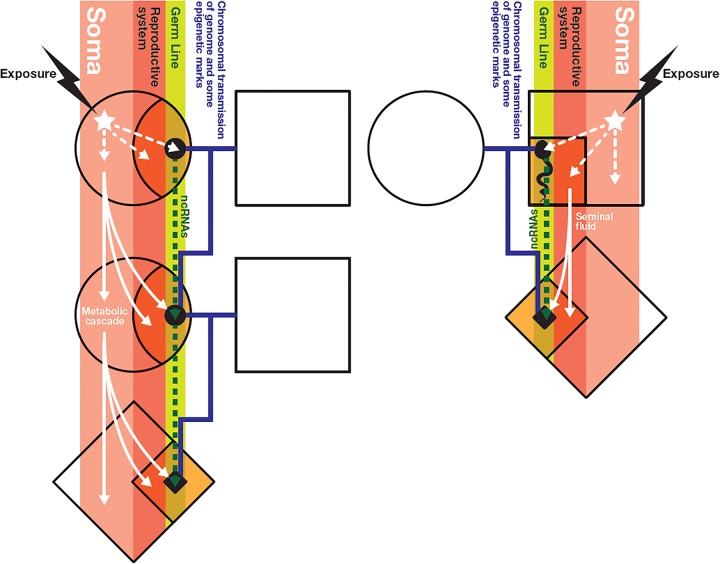
Schematic pedigree diagram showing the main routes for biological transmission of the effects of exposure to the next generation(s). Left, female line; right, male line. The exposure can potentially affect the germline, the reproductive system and the soma more generally. The traditional pedigree lines (blue) show chromosomal transmission, which, in addition to the DNA, can potentially include exposure-induced epigenetic marks that escape erasure and impact on offspring development. The germline can potentially transmit exposure-induced non-coding RNAs (ncRNAs) that influence offspring development. Exposure induced metabolic changes can set up a ‘metabolic cascade’ such that changes in the reproductive tract influence early embryo programming of the offspring or change metabolic signals across the placenta. An additional maternal transmission route is the influence of the mother's microbiome on that of her child.

In contrast, biological inheritance of phenotype from one generation to the next is usually regarded as resulting from transmission of genetic material (DNA) from both parents, plus ‘maternal effects’ either (i) carried within the egg cytoplasm (such as in mitochondria, proteins and RNA molecules), (ii) experienced in utero, primarily through the transplacental passage of nutrients, metabolic signals and toxins, or (iii) experienced early postnatally, via breast feeding and/or the exchange of microbiota. Additionally, the maternal genotype also contributes to the early nutritional environment of progeny through its influence on maternal metabolism during pregnancy. This is best exemplified in studies on insulin sensing and fetal growth.^6^ It is plausible that a maternal exposure in pregnancy could induce a ‘metabolic cascade’ to subsequent generations, whereby fetal programming could alter later adult metabolism, which, in turn, changes the physiology of the uterus receiving and programming the early embryo of the next generation^7^ and/or the transplacental metabolic signals to the fetus. Theoretically this process could be genotype independent. The challenges of unravelling the complexities associated with maternal transmission of exposure effects are manifold due to the multiple levels of interdependence (figure 1).

Until recently, paternal exposures prior to conception (other than those known to induce DNA mutations) were assumed to have little or no effect on offspring development, primarily due to the lack of a plausible biological mechanism. So ingrained was this view that confirming a lack of paternal exposure effect has been promoted as a useful ‘control’ for social confounders in the study of maternal exposure effects.^8^ ^9^ However, several robust animal experiments exist showing transgenerational phenotypic effects of exposures via the male line.^10–16^ Human observational studies (reviewed in^17^) also support this type of non-genetic transmission.

In light of the emerging evidence for both maternal and paternal transmission of non-genetic, non-cultural effects to progeny, consideration must be given to extend traditional epidemiological methodology to include not only early-life paternal and maternal exposures, but also ancestral exposure data. The consequences of such transmission are potentially profound, particularly in relation to past assumptions underpinning much of epidemiological and genetic analyses. In transgenerational epidemiology, assuming cultural inheritance automatically when results make genetic inheritance implausible (and vice versa) is no longer acceptable.

### Framing future transgenerational epidemiological research

Although some have adopted the terms *intergenerational*, *multigenerational* and *transgenerational* to mean specific routes and numbers of generations of transmission,^18^ we use ‘transgenerational’ in a general sense to describe a measurable outcome in one generation (offspring) with exposures in either the mother or the father (prior to conception), or their respective parents or ancestors. ‘Transgenerational’ was first used in this context in the 1980s for the apparent transmission of the trauma suffered by Holocaust survivors to their unexposed offspring.^19^ This term was also adopted in early speculation about imprinted genes mediating the transmission of exposure information to the next generation^20^ and is used in recent reviews of the subject in mammals.^15^ Importantly, the mechanism of transmission must be primarily non-genetic, although mounting evidence implicates genetic factors as moderators of such effects (see below). In prenatal exposure via the mother, the fetus can be assumed to be directly exposed; and here the outcomes of interest in a ‘transgenerational’ context would be in that child's own offspring.

A priority for transgenerational epidemiology is to describe the range of measurable exposures and outcomes mediating transgenerational effects in humans. That is, *what* exposure at *which life stage* in parents, grandparents or distant ancestors is associated with a measurable phenotypic outcome in the offspring or subsequent generations? We suggest this observational research is best done in a setting where the effects of social patterning can be accounted for, thereby distinguishing cultural inheritance from a more direct biological transmission across generations. Defining exposure-sensitive periods associated with transgenerational effects will be highly informative in this regard.

Unlike much of the research in the field to date, it is important that future transgenerational epidemiology should not be restricted to the inheritance of acquired characteristics, or ‘phenotypic transmission’ to the next generation. The *exposure* is key, not the founder, phenotype. In fact, there need not be a measureable phenotype in the exposed (or intermediate) generation. Similarly, phenotypic outcomes should not only be assumed to be detrimental. Although the usual research design in transgenerational epidemiology is to test associations between parental or ancestral exposure and adverse outcomes in *unexposed* offspring, transgenerational responses/effects can also be protective ‘adaptations’, potentially lessening the risk of adverse outcomes in successive *exposed* generations. The latter has been demonstrated in a remarkable male line rat experiment where ancestral carbon tetrachloride-induced liver damage led to heritable, epigenetically mediated, reprogramming of hepatic healing, such that exposure in the third generation did not produce liver cirrhosis.^21^ Lastly, the exposures associated with transgenerational effects need not be adverse. Enriched environments can induce transgenerational responses in mice.^22^

In summary, a transgenerational perspective is needed if we are to better understand the determinants of major public health problems and adequately assess the value and feasibility of potential interventions. Studies in the field should not be predicated upon a particular mode of transmission from one generation to the next and should make no assumptions about the relative contributions of cultural, genetic or other molecular mechanisms of inheritance. We need to keep an open mind and design appropriate experiments for testing each potential mode of transmission independently.

## Human observations

Below we summarise human studies by type of exposure and route (paternal or maternal) of transmission. Studies of exposures around the time of conception^23–26^ are *not* included.

### Food supply

#### Paternal line

Compelling historical findings of paternal transgenerational effects in humans come from the Överkalix population in northern Sweden^27–31^ using samples of individuals born in specified years. Longevity and specific causes of death were linked to detailed historical records of harvests and food supply experienced by previous generations in early life. This series of studies is summarised in table 1. The initial all-cause mortality study^27^ noted an exposure-sensitive period during mid-childhood (the 'slow growth' period during the few years leading up to the prepubertal growth spurt) but not later in childhood, which was confirmed in a subsequent analysis in 2 of 3 independent cohorts^28^ and then in a sex-specific analysis of mortality rate in the grandchildren.^29^ This latter analysis showed mortality rate of men born in the target years was linked to just their paternal *grandfather's* food supply in mid-childhood, whereas the mortality rate of the women studied was associated solely with the paternal *grandmother's* food supply. The mortality rate ratio point estimates of grandsons were 1.67 or 0.65 depending on good or poor food supply of the paternal grandfather during the slow growth period (compared with grandsons of grandfathers with moderate food supply). For granddaughters, the comparable figures were 2.13 and 0.72 for good or poor food supply of the paternal grandmother. This intriguing pattern of sex-specific association persisted when the grandchild's early life circumstances were taken into account.^30^ A recent study^31^ shows that sudden change in food supply between 0 and 13 years of the paternal grandmother is linked to cardiac deaths in the granddaughters raising the possibility that this type of exposure might underlie some of the transgenerational responses reported earlier. Recently, a discussion paper has been posted^32^ that used data on the German famine of 1916–1918 to look at height, mental health and educational achievement in the descendants of boys exposed at 9–12 and girls at 8–10 years. Among the third generation, males tend to have better mental health scores if their paternal grandfather was exposed, thus providing support for the importance of exposures in the mid-childhood period.

**Table 1:** Summary of the historical transgenerational studies from Överkalix, Northern Sweden

Reference	Överkalix cohorts by grandchild's or proband birth year	Prior research question	Main findings	Comments
Bygren *et al*^27^	1905 (n=94)	Any link between ancestral food supply at two periods in childhood, the prepubertal spurt or the period just before and proband *longevity*?	*Paternal grandfather*'s food supply just *before prepubertal growth spurt* inversely associated with proband longevity	This study defined the mid-childhood ‘slow growth period’ as an exposure period associated with transgenerational effects
Kaati *et al*^28^	1895 (n=107)	Any link between ancestral *mid-childhood* food supply and proband *cardiovascular and diabetes mortality*?	Father's poor, and mother's good, food supply in mid-childhood linked to *reduced proband cardiovascular mortality*. Paternal grandfather's good mid-childhood food supply linked to *increased proband diabetic mortality*	Diabetic mortality was included as a prior hypothesis based on possible role of imprinted genes. Each diabetic proband had a different paternal grandfather (Bygren *et al.* 2006)
Bygren *et al*^99^ ^100^	1905 (n=99)			
	1920 (n=111)			
Pembrey *et al*^29^	1895 (n=107)	Any *sex-specific* link between (grand) parental mid-childhood food supply and proband *mortality rate ratio*?	*Paternal grandfather’*s food supply linked to *grandson’*s mortality; *paternal grandmother's* food supply to *granddaughter's* mortality	Stratification by sex of the proband suggested by early ALSPAC results of paternal smoking effects (Northstone *et al.* 2014^39^). Exposure-sensitive period in mid childhood but not (pre)puberty confirmed
	1905 (n=99)			
	1920 (n=111)			
Kaati *et al*^30^	1895 (n=107)	Any link between (grand) parental mid-childhood food supply and proband *early-life circumstances and sex-specific longevity*?	Grandparental sex-specific transgenerational effects (as above) persisted. Parental effects now revealed as well	Taking proband's early-life circumstances into account revealed a father to son effect on longevity
	1905 (n=99)			
	1920 (n=111)			
Bygren *et al*^31^	1895 (n=107)	Any link between *sharp change* in grandparental food supply in childhood (0—13ys) and proband *cardiovascular mortality*?	Sharp change in food supply of *paternal grandmother* linked to increased cardiovascular mortality in *female* probands	Prior hypothesis—transgenerational effects of change in supply as the demonstrated effects from gestation to adulthood (Bygren *et al.* 2000)^101^
	1905 (n=99)			
	1920 (n=111)			

#### Maternal line

Human studies of ancestral famine or food supplements during pregnancy on fetal growth of the grandchildren have been recently reviewed.^18^ The Guatemalan follow-up studies of the descendants of women with nutrition intervention in pregnancy indicated a positive association between improved pregnancy and first-generation progeny nutrition with grandchild development indicators—primarily higher birth weight and increased length.^33^ However, the findings were complicated by supplement use in pregnancy and breast feeding. Cross-sectional studies of the famine in China showed women exposed in utero and early life had bigger babies.^34^ A far more precise definition of famine was available in the widely cited Dutch famine studies. At the end of World War II (1944–1945), acute food shortages caused widespread starvation (the Hunger Winter) in the occupied cities of Holland. The first study of multigenerational outcomes concerned birth weights of 1808 firstborn children to mothers, most of them exposed in utero during the famine.^35^ The birth weights of babies born to women who were exposed to famine in utero (during the first and second trimesters) were lower than babies of non-exposed women. Subsequent prospective studies revealed a more complicated situation; in essence, the first born of women exposed in utero were heavier than controls, while second and third born were lighter at birth, the reverse of what is normally observed.^36^ ^37^ In other analyses, no association was found between prenatal famine exposure and grandchild birth weight, whereas associations were identified between exposure and childhood adiposity in grandchildren.^38^

### Smoking

#### Paternal line

The study of the age of *onset* of paternal smoking on offspring outcomes (study child), using the Avon Longitudinal Study of Parents and Children (ALSPAC), was designed to specifically test the Överkalix hypothesis that mid-childhood was an exposure-sensitive period with respect to transgenerational effects. The initial offspring outcomes were those relevant to the Swedish results such as birth characteristics and body mass index (BMI) at ages 7 and 9 years. ALSPAC data, adjusted for paternal smoking at conception, showed that the earlier the father started smoking, the greater the BMI at 9 years of sons, but not daughters. The greatest association was found in sons of men who commenced smoking before the age of 11.^29^ Follow-up to age 17 years has just been reported.^39^ The adjusted mean differences in BMI, waist circumference and total fat mass in the group of sons whose fathers started smoking <11 years, relative to others, increased with age, being significantly greater (eg, an extra 5–10 kg of fat mass) from 13 years onwards. There was no significant BMI association in daughters, but they showed a reduction in total lean mass.

#### Maternal line

An ALSPAC study of non-smoking ALSPAC mothers, who were exposed in utero to maternal smoking, showed their sons (but not daughters) to be, on average, 61 g bigger at birth compared with babies where neither the study mother or grandmother smoked.^40^ There were no fetal growth differences if the father's mother had smoked in pregnancy. Misra and colleagues analysed data on maternal and grand-maternal smoking from the Baltimore branch of the US Collaborative Perinatal Project and after many adjustments found there was a 244 g increased weight in offspring of non-smoking mothers if their own mother had smoked prenatally.^41^ Similarly, in a study of births to non-smoking women participating in the British 1958 birth cohort, after adjustment for gestation, maternal birth weight, maternal height and BMI, there was a mean increase of 45 g in weight in children of mothers exposed in utero to maternal smoking*,* but no allowance was made for parity.^42^ Neither of these analyses assessed whether there were different effects according to gender of the offspring.

In addition to studies examining anthropometric measures, a few studies have also examined the transgenerational effects of smoking on asthma. One much-quoted study published in 2005 indicated that the risk of childhood asthma was not only influenced by prenatal maternal smoking, but also by the exposure of the mother in utero to her own mother's smoking.^43^ The ALSPAC study did not find an effect of maternal exposure in utero on childhood asthma,^44^ although there was suggestive evidence of paternal prenatal exposure being associated with asthma and persistent wheezing in his daughters. Both findings therefore need to be replicated.

### Other paternal line exposures

#### Betel nut

An experimental study of betel nut (*Areca catechu*) in CD1 mice indicated that paternal exposure transmitted an increased risk of hyperglycaemia and obesity to non-betel-fed first-generation offspring, especially males.^45^ This led to an observation study within the Keelung Community-based Integrated Screening Program in Taiwan studying the effect of paternal betel-quid (*paan*) chewing prior to offspring conception. Chewing usually began around 18 years (Boucher, personal communication) and interestingly, a similar association to that found in mice was observed, with a dose-dependent association of paternal betel-quid use with early metabolic syndrome in the adult offspring who had never chewed betel-quid themselves.^46^

### Conclusions from human observations to date

It is fair to say that publicity related to recent publications^31^ ^39^ has drawn some statistical criticism on the issue of multiple testing. Our response points to there being clear prior hypotheses (see table 1) and coherence between different studies/populations. We recognise that research conclusions that extend the public health paradigm need a higher level of evidence than normal, and further replication is needed. However, taken together the above studies demonstrate transgenerational associations between grandpaternal/paternal exposures and health outcomes that are most unlikely to be due to ‘cultural’ inheritance/social patterning or genetic inheritance in isolation. The demonstration of an exposure-sensitive period in mid-childhood prior to puberty, but not in adolescence, provides compelling evidence for some other form of transmission between generations.

## Lessons from animal experiments

This is not the place to review the large number of mammalian experiments characterising transgenerational responses to ancestral exposures. Nevertheless, such experiments have revealed certain features that need to be considered in designing and interpreting human studies.

### Sex differences in transgenerational effects

Sex differences occur in most non-communicable diseases, including metabolic diseases, diabetes, hypertension, cardiovascular disease, psychiatric and neurological disorders and cancer, yet the potential role of non-genetic transgenerational effects in mediating this has rarely been investigated.

Mammalian experiments showing transgenerational responses have reported numerous sex-specific effects after exposure during pregnancy or on paternal exposure before breeding. These can affect offspring of both sexes,^14^ ^45^ solely/predominantly males^47^ ^48^ or solely/predominantly females.^12^ Thus, in line with the Överkalix results, the phenotypic outcomes are generally not sex-limited. The X and Y chromosomal difference between the sexes could potentially contribute to sex-specific transgenerational effects. This could include hormonal effects as well as effects that are independent of sex steroids. A recent review of sexual dimorphism^49^ emphasises the differential epigenetic processes in the placenta, noting the abundance of X-linked genes involved in placentogenesis and the early unequal gene expression by the sex chromosomes between males and females. Champagne's comment on sex specificity in transgenerational responses highlights the differences in epigenetic plasticity during gamete maturation.^50^ The large number of rodent examples of sex differences in outcomes indicates that the sex differences in outcomes observed in the few human studies (summarised above) are not unexpected. Despite this, such differences tend to be viewed with suspicion, perhaps because of the dangers of *post-hoc* subgroup analysis.^51^ However, with due attention to testing for gender interaction as part of association studies, these sex-specific effects should inform the search for mediating mechanisms in transgenerational responses.

The combination of sex-specific transmissions and outcomes in the Överkalix study are consistent with X and Y chromosome transmission and have led to hypotheses in which the non-recombining part of the Y chromosome could more easily retain epigenetic marks in the gametes^52^ or carry a genomic stress ‘sensor’ where DNA damage triggers a non-coding RNA response.^17^ However, it should be noted that some sex-specific transgenerational effects in mice are incompatible with XY transmission.^50^ ^53^ Nevertheless, it is highly plausible that transgenerational responses are one of the determinants of common non-communicable diseases and also of some of the sex differences. Only a transgenerational extension of epidemiological research can address this question.

### Variable outcomes across the generations from ancestral exposure

Interestingly, the phenotypic effect of an ancestral exposure can vary across generations. This may reflect a propensity for one sex to transmit but not manifest the transgenerational effect.^53^ Using unpredictable maternal absence in neonatal mice as a stress-inducing exposure, Franklin *et al* found transgenerational effects through the male line that included transmission by intermediate males who were not themselves overtly affected.^54^ This model has been explored further in terms of sperm non-coding RNAs^55^ (see below). In contrast, other studies have shown that transgenerational effects may become restricted over multiple generations. For example, in a study of maternal high-fat diet exposure in mice, there was an increase in body size and reduced insulin sensitivity that persisted across two generations via both maternal and paternal lineages. However, an examination of third-generation progeny revealed that only females displayed the increased body size phenotype, and this effect was only transmitted via the paternal lineage.^56^ As highlighted by these authors, an emerging phenomenon is that some traits extinguish via one or both lineages, whereas others persist across generations, suggesting that divergent mechanisms of transmission may be involved at the same time.

The majority of animal experiments have tended to restrict phenotyping of descendants to the system that was disturbed by the ancestral exposure, for example, dietary exposures with metabolic dysfunction, stress with behavioural outcomes. Given our present state of ignorance, such limited analysis is unwise. Most human multigenerational cohorts have a range of measures that would allow studies to address the question of whether outcomes in descendants are limited to the system being challenged by the exposures or can be of a more generic character. Interestingly, a recent mouse study using a stress-inducing exposure found both behavioural and metabolic disturbances in descendants.^55^

## Possible mediating molecular mechanisms

A true transgenerational effect has several steps:—exposure; the biological embedding or ‘capture’ of the exposure (this may include DNA damage/mutation); transmission of this information between generations; and phenotypic change in (unexposed) offspring/descendants, potentially associated with fetal programming*.* The mediating mechanism may vary between steps/generations and therefore may require independent investigation as part of an epidemiological framework.

### Transmission between generations

As noted above (figure 1), transmission of exposure information down the maternal line may have several different routes with potential to confound any specific mechanism. Thus, proof-of-principle studies often focus on the male line, even if the initial exposure is during fetal development via the pregnant mother/dam. In the latter situation, the exposed pregnant individual is usually termed F0.

Irrespective of the exposure type and timing, a prerequisite for transgenerational effects beyond the second generation is the involvement of germline transmission, which may occur in several ways. First, by directly impacting on the developing primordial germ cells early in utero, as has been reported in mice.^57^ In this instance, resulting gametes produced later in life might be anticipated to carry a transmissible ‘memory’ of the original exposure via the mother that can be passed on to subsequent generations. Alternatively, any effect transmitted to the F2 generation via F1 gametes might arise purely in response to the altered development or metabolism of the F1 generation arising from the F0 exposure (see Aiken and Ozanne^7^ for a discussion of such ‘propagational programming’). These are two distinct processes, each of which might mediate the effects of an F0 exposure on the F2 and subsequent generations, independently of non-gametic maternal effects. It is worth noting that even male gametic transmission does not necessarily require the molecular ‘memory’ of the original exposure to pass through meiosis. There are tight cell–cell junctions between Sertoli-cells and postmeiotic spermatids that theoretically could act as a route for ‘message delivery’ to the zygote and beyond,^58^ and recent work has shown an influence of seminal fluid in conjunction with uterine factors on offspring metabolic development.^59^

### A role for epigenetics in transgenerational responses

Epigenetic events are an essential mediator of cellular differentiation and therefore development in multicellular organisms. However, the underlying dynamism and responsiveness to subtle subcellular cues that are a hallmark of epigenetic change during development also make the developing epigenetic profile susceptible to external influence, with the potential to ‘program’ the underlying genome towards an altered phenotype in later life.^60^ The labile nature of some epigenetic marks to environmental influence has made epigenetic processes an appealing mechanism for transgenerational responses. In fact, transgenerational epigenetic inheritance has been widely documented in prokaryotes, fungi, plants and animals, via both the male and female germline. In at least some instances (eg, in *Caenorhabditis elegans*), initial transmission down the female line can ‘spread’ to encompass paternal transmission in subsequent generations.^61^

In mammals, data demonstrating the erasure of nearly all epigenetic marks twice during the life course (early postzygotically and in the developing primordial germ cells in early development) have been difficult to reconcile with an epigenetic mode of transgenerational effects. Nevertheless, for decades it has been known that mammals have a set of imprinted genes; those that are selectively silenced in either the maternal or paternal germline during gamete formation, such that only one allele is expression following fertilisation. Genomic imprinting establishes the principle of transgenerational epigenetic inheritance—the gene is active or silent depending on epigenetic marks placed in the parental generation that survive erasure as they pass to the offspring.^62^ The parent-of-origin-dependent gene expression reflects a robust, evolved response to differences in cellular conditions between egg and sperm development. Imprinted genes escape one of the two phases of genome demethylation between generations, namely in the preimplantation embryo soon after fertilisation.^63^ Importantly, the epigenetic marks that regulate some imprinting regions have been demonstrated to show interindividual, tissue-specific, age-specific and environmentally sensitive variation.^25^ ^64–72^

Recent mouse experiments have also reported a small percentage of loci that escape demethylation at the second phase during primordial gonadal cell differentiation in the early embryo. These are largely associated with repeat sequences, often adjacent to transposable DNA elements.^73^ Interestingly, there is growing evidence that some repeat-based transposable elements have gene enhancer activity.^74^ It is also now clear that many non-imprinted, non-repetitive genomic loci remain subject to epigenetic control in both sperm and eggs. This applies to both DNA methylation^75^ ^76^ and histone modification profiles.^77^ ^78^ Further, emerging data exist that demonstrate intergametic variation in epigenetic profile, supporting a role for environmental and/or genetic influence in determining the epigenetic state transmitted to the next generation.^79^ ^80^

Of particular note is a recent study in mice that provides a plausible mechanism for transgenerational inheritance of environmental information, linking behavioural, neuroanatomical and epigenetic processes. Using an olfactory fear conditioning protocol, wherein an odour (acetophenone) is paired with a mild foot-shock, Dias *et al* noted a behavioural sensitivity to acetophenone in the next two generations of animals, despite having no exposure to this odour. This was associated with structural changes in the olfactory nervous system accompanying the fear conditioning and distinct hypomethylation of the *Olfr151* gene (known to be activated by acetophenone) in sperm of conditioned F0 males and F1-naive offspring.^16^ The extent to which other specific environmental exposures elicit similarly specific epigenetic changes in gametes, and the extent to which these are retained during postzygotic development, remains unclear but warrants further investigation.

Another recent study in a non-genetic prediabetes mouse model provides further compelling evidence for gametic transmission of environmentally induced epigenetic change.^81^ In this model, offspring of prediabetic fathers show glucose intolerance and insulin resistance in association with distinct changes in gene expression and DNA methylation patterns in pancreatic islets, including reproducible changes in methylation of insulin signalling genes. Interestingly, the sperm of prediabetic fathers have extensive changes in methylation patterns in sperm, many of which overlapped with that of pancreatic islets in offspring.^81^

### Non-coding RNAs (ncRNAs) as mediators of transgenerational responses

There is evidence for a role of sperm-derived RNAs in mediating paternal transgenerational effects, with several classes of RNA recently identified in sperm that are likely to be essential factors in male fertility, including many Y-linked ncRNAs.^82^ Such RNAs have the potential to play roles in early postfertilisation development.^83^

The specific RNA content of sperm has been assessed in response to specific environmental exposures. Diet-induced paternal obesity in mice alters microRNA (miRNA) content and sperm methylation status in male mice in association with altered metabolic function in two subsequent generations of progeny.^84^ Chronic stress in male mice results in altered miRNA content of sperm in association with reduced HPA stress axis responsiveness in the progeny.^85^ Recently, exploiting their transgenerational model of unpredictable maternal absence in neonatal mice as a stress-inducing exposure, Mansuy's group have examined the associated miRNA changes in sperm. Injection of sperm RNAs from exposed males into fertilised wild-type oocytes reproduced the behavioural and metabolic alterations in the resulting offspring. Interestingly, affected F2 mice had normal sperm miRNAs, yet their F3 offspring still inherited the behavioural phenotype.^55^ In humans, the small RNA component of sperm is altered in male smokers, including miRNAs predicted to target the epigenetic regulators.^86^ As a whole, the emerging picture suggests that the RNA content of sperm is sensitive to environmental exposures, with the potential to influence early embryonic development, including the establishment of the postzygotic epigenome. This is speculated to be an initiating mechanism for transmission of impaired metabolic health to future generations via the male lineage.^84^

### A modifying role for genetics in mediating transgenerational effects

Genetic variation will clearly impact to a variable extent on the apparent heritability of some epigenetic marks as evidenced by multigenerational familial studies^87^ and exemplified at one extreme by *MLH1* germline epimutation analysis. Originally thought to be solely due to inheritance of a ‘pure’ epigenetic variant (DNA hypermethylation) across generations,^88^ in some cases this is genetically driven, being erased in the germline but faithfully re-established early postzygotically in association with a proximal genetic variant or haplotype.^89^ ^90^ Epigenetic responses to exposures and the propensity to be transmitted to the next generation(s) are likely to have evolved under selective pressure. As such one can expect variation in ‘responsiveness’ to a specific exposure to be underpinned by genetic differences. Compelling data in support of this have recently emerged, highlighting the potential of genetic variation in both *cis* (regions on the same chromosome) and *trans* (different chromosome) to mediate the effects of environmental exposures on the neonatal epigenetic profile in humans.^91^ As such, any study of environmentally driven epigenetic variation transmitted transgenerationally needs to considered on a background of genetic variation as a modifier of this effect. For example, although genetic differences may contribute directly to the phenotype in a specific environment, in a classic G×E manner, the additional layer of epigenetic regulation has the potential to make such effects more nuanced on certain genetic backgrounds. Alternatively, some transgenerational environmentally induced epigenetically mediated effects may be exacerbated in association with other ‘risk’ genotypes. Finally, some transgenerational phenotypic effects are likely to be completely independent of genotype.

### Different transmitting mechanisms within the same lineage

As described above, environmental exposures can potentially initiate transgenerational effects through altered miRNA in sperm, but some aspects of the phenotype can be transmitted to later generations in the absence of the original miRNA change.^55^ This separation of initiation and later transmission has been explored using experimental genetic changes. For example, experimental genetic effects in rodents can set up a developmental environment that promotes transgenerational responses in the absence of the genetic change, for example, by changing weight and food intake in subsequent generations.^92^ It is plausible that ‘random’ somatic mutations leading to substantial mosaicism might induce epigenetic or ncRNA changes that in turn initiate transgenerational effects, or exposures can induce DNA damage as the first step, for example, stress-related DNA damage.^93^ Further, recent data in mice have revealed that genetic variation in the genes regulating one carbon metabolism can manifest as distinct and reproducible phenotypic variation across multiple systems and generations.^94^ Importantly, these defects were dependent upon the genotypes of the maternal grandparents. Embryo transfer experiments revealed that haploinsufficiency in mice leads to two distinct, separable phenotypes: adverse effects on their wild-type daughters' uterine environment, leading to growth defects in wild-type grandprogeny, and the appearance of congenital malformations independent of maternal environment that persist for five generations, likely through transgenerational epigenetic inheritance.^94^

## What is needed for future human studies?

Non-genetic biological transmission between generations, of which epigenetic hypotheses are an exemplar, spell out how exposures in one generation may lead to effects on health, behaviour or other personal characteristics in later generations. To date human studies of transgenerational responses have ranged from historical analysis of the Överkalix cohorts for which biological samples are not available (at least for all but the 1935 cohort) to the contemporary birth cohort ALSPAC where samples are available on the mother and offspring, plus limited paternal samples. The collection of blood or tissue samples is often seen as the next step to further our knowledge about transgenerational mechanisms in humans. Discoveries of epigenetic and other mechanisms in cells may indeed further our understanding of the aetiological relations. However, this does not mean that large population studies without direct information from human blood or tissue are redundant. On the contrary, epigenetic hypotheses that predict that a specific exposure in one generation will give rise to a specific outcome in a later generation(s) could and should be tested in large-scale population studies that hold information about such exposures and outcomes. Conventional non-experimental epidemiological studies can still provide an effective means to explain and potentially prevent (adverse) transgenerational responses. Public health interventions may be applied at many different entry points in the aetiological chain of events. Often one and the same condition may be causally attributed to different aetiological antecedents in the web of causation dependent on whether the focus of the study is primarily preventive, predictive, therapeutic or, more generally, explanatory.^95^ ^96^

There are many birth cohorts and other general population samples worldwide (table 2), and current life-course epidemiology is well placed to extend its reach beyond a single generation and the DNA sequence. It is important to note that from the public health perspective, studying the effect of just early-life parental exposures is likely to increase our understanding of the phenotypic variation, health and well-being of the offspring generation, and perhaps point the way to intervention trials. Common parental exposure information is likely to be available, or could be collected, in many of the existing cohorts. Going forward, the primary aim of human studies may not be to test a *particular* mechanistic theory, although studies over three generations with biological samples are likely to be informative in this respect. However, it will be important to pay particular attention to the parental/ancestral exposures, both in terms of dose and timing of the exposure in relation to the person's stage of development—ideally with prospectively collected information. Standardisation of outcome measures across cohorts is important for many epidemiological studies, including transgenerational studies, and is being actively addressed.

**Table 2:** Additional examples of birth cohorts with the capacity to explore non-genetic transgenerational effects in humans (see text)

Cohort name	Participant number*	General description	Reference
ABC	106 370	The Aarhas Birth cohort Denmark has collected data during pregnancy and delivery for women since 1989. The associated biobank was established in 2008 to provide the opportunity to investigate the role of genetic factors, environmental exposures and lifestyles in pregnancy on the risk of disease in the offspring.	102
ABIS	17 045	All Babies in Southeast Sweden (ABIS) is a retrospective birth cohort of 17 055 children born October 1997–October 1999. Parental and child questionnaire follow-up. Extensive biobank with repeated sampling at follow up.	†
ALSPAC	14 541	The Avon Longitudinal Study of Parents and Children recruited 14 541 pregnant women with due date April 1991–December 1992. Information on parents’ life taken during study pregnancy. Mothers and children have been followed using questionnaires. Children have been followed at regular clinical assessment visits. Extensive biobank.	103
BCS70	17 000	The British Cohort Study follows people born in the UK in a single week of 1970. It has collected information on health, physical, educational and social development, among other factors.	104
BIB	13 776	Born in Bradford is a longitudinal birth cohort study aimed at recruit a multiethnic cohort of babies born in Bradford (UK) and their parents in order to investigate fetal growth, birth and long-term outcomes by ethnic groups.	105
DNBC	94 837	The Danish National Birth Cohort recruited pregnant women and their children from 1996 to 2002. Multiple interview data and biospecimens were collected including food frequency questionnaire in gestational week 24 and periconceptional use of medicine and food supplements. Data for the cohort are collected at regular times from hospital discharge registry and other national registers.	106
MCS	19 000	The Millennium Cohort Study follows the lives of children born in the UK in 2000–2001. It collects information on siblings and parents covering topics including socioeconomic variables, behaviour and cognitive development**.**	107
MOBA	108 500	The Norwegian Mothers and Babies study aims to quantify the influence of various social, genetic, nutritional and environmental exposures on pregnancy outcomes and child health. Data and biospecimens were collected in pregnancy and at birth. Fathers were also recruited and provided blood. Health outcomes were collected from hospital discharge registries as well as other health registries such as the Medical Birth Registry, the Cancer Registry and the Diabetes Registry.	108
NCDS	17 000	The National Child Development Study (1958 birth cohort study) follows the lives of children born in the UK in a single week of 1958. It has collected information on physical and educational development, economic circumstances, employment, family life and health behaviour among other variables. DNA bank.	109
UBCoS Multigen	7567	Uppsala Birth Cohort Multigenerational Study exploring several issues highly relevant for health equity research. Life-course approach to analysis detailed biological and social data stretching from birth to old age with access to more than two successive generations.	110

*Only those with >10 000 participants or a focus on multigenerational studies are included.

†http://www.abis-studien.se/hem/english-11100423. Further information from the European cohort available at http://www.birthcohorts.net/.

### Biological samples

Many cohorts are establishing DNA banks, but it is also worth bearing in mind that neonatal blood spots (Guthrie cards), collected and stored for many decades in some countries, can be a valuable resource for capturing neonatal epigenome and other data.^97^ In light of the role of miRNAs, discussed above, the collection of cell-free circulating miRNAs is likely to be a valuable additional resource.^98^ In terms of ‘future proofing’, serious consideration should be given to storing ‘living genomes’ in the form of EBV-transformed lymphoblastoid cell lines or other viable cells derived from primary tissues (eg, induced pluripotent stem cells). The value of such cells in assessing epigenome associations with early-life experience is an active research area, including how to deal with possible culture-induced genetic and epigenetic changes. If such approaches prove useful, this will offer considerable opportunities for life course and transgenerational epidemiology.

## References

[1] Forsdahl A Are poor living conditions in childhood and adolescence an important risk factor for arteriosclerotic heart disease? Br J Prev Soc Med 1977;31:91–5 884401 10.1136/jech.31.2.91 PMC479002

[2] Barker DJ The fetal and infant origins of adult disease. BMJ 1990;301:1111 2252919 10.1136/bmj.301.6761.1111 PMC1664286

[3] Barouki R Gluckman PD Grandjean P Hanson M Heindel JJ Developmental origins of non-communicable disease: implications for research and public health. Environ Health 2012;11:42 22715989 10.1186/1476-069X-11-42 PMC3384466

[4] Modin B Vågerö D Hallqvist J Koupil I The contribution of parental and grandparental childhood social disadvantage to circulatory disease diagnosis in young Swedish men. Soc Sci Med 2008;66:822–34 18155818 10.1016/j.socscimed.2007.11.001

[5] Modin B Erickson RP Vågerö D Intergenerational continuity in school performance: do grandparents matter? Eur Soc Rev 2013;29:858–70

[6] Weedon MN Clark VJ Qian Y Ben-Shlomo Y Timpson N Ebrahim S Lawlor DA Pembrey ME Ring S Wilkin TJ Voss LD Jeffery AN Metcalf B Ferrucci L Corsi AM Murray A Melzer D Knight B Shields B Smith GD Hattersley AT Di Rienzo A Frayling TM A common haplotype of the glucokinase gene alters fasting glucose and birth weight: association in six studies and population-genetics analyses. Am J Hum Genet 2006;79:991–1001 17186458 10.1086/509517 PMC1698701

[7] Aiken CE Ozanne SE Transgenerational developmental programming. Hum Reprod Update 2014;20:63–75 24082037 10.1093/humupd/dmt043

[8] Smith GD Assessing intrauterine influences on offspring health outcomes: can epidemiological studies yield robust findings? Basic Clin Pharmacol Toxicol 2008;102:245–56 18226080 10.1111/j.1742-7843.2007.00191.x

[9] Macdonald-Wallis C Tobias JH Davey Smith G Lawlor DA Parental smoking during pregnancy and offspring bone mass at age 10 years: findings from a prospective birth cohort. Osteoporos Int 2011;22:1809–19 20967424 10.1007/s00198-010-1415-y PMC3092913

[10] Anway MD Cupp AS Uzumcu M Skinner MK Epigenetic transgenerational actions of endocrine disruptors and male fertility. Science (New York, NY) 2005;308:1466–9 10.1126/science.1108190 PMC11423801 15933200

[11] Franklin TB Russig H Weiss IC Graff J Linder N Michalon A Vizi S Mansuy IM Epigenetic Transmission of the Impact of Early Stress Across Generations. Biol Psychiatry 2010;68:408–15 20673872 10.1016/j.biopsych.2010.05.036

[12] Ng SF Lin RC Laybutt DR Barres R Owens JA Morris MJ Chronic high-fat diet in fathers programs beta-cell dysfunction in female rat offspring. Nature 2010;467:963–6 20962845 10.1038/nature09491

[13] Burdge GC Slater-Jefferies J Torrens C Phillips ES Hanson MA Lillycrop KA Dietary protein restriction of pregnant rats in the F0 generation induces altered methylation of hepatic gene promoters in the adult male offspring in the F1 and F2 generations. Br J Nutr 2007;97:435–9 17313703 10.1017/S0007114507352392 PMC2211514

[14] Carone BR Fauquier L Habib N Shea JM Hart CE Li R Bock C Li C Gu H Zamore PD Meissner A Weng Z Hofmann HA Friedman N Rando OJ Paternally induced transgenerational environmental reprogramming of metabolic gene expression in mammals. Cell 2010;143:1084–96 21183072 10.1016/j.cell.2010.12.008 PMC3039484

[15] Daxinger L Whitelaw E Understanding transgenerational epigenetic inheritance via the gametes in mammals. Nat Rev 2012;13:153–62 10.1038/nrg3188 22290458

[16] Dias BG Ressler KJ Parental olfactory experience influences behavior and neural structure in subsequent generations. Nat Neurosci 2014;17:89–96 24292232 10.1038/nn.3594 PMC3923835

[17] Pembrey ME Bygren LO Golding J The nature of human transgenerational responses. In: Jirtle HJ Tyson FL, ed. Environmental Epigenomics in Health and Disease Epigenetics and Disease Origins. Heidelberg: Springer, 2013:257–1.

[18] Susser E Kirkbride JB Heijmans BT Kresovich JK Lumey LH Stein AD Maternal prenatal nutrition and health in grandchildren and subsequent generations. Annu Rev Anthropol 2012;41:577–610

[19] Nadler A Kav-Venaki S Gleitman B Transgenerational effects of the holocaust: externalization of aggression in second generation of holocaust survivors. J Consult Clin Psychol 1985;53:365–9 4008720 10.1037//0022-006x.53.3.365

[20] Pembrey M Imprinting and transgenerational modulation of gene expression; human growth as a model. Acta Geneticae Medicae Et Gemellologiae 1996;45:111–25 8872020 10.1017/s0001566000001197

[21] Zeybel M Hardy T Wong YK Mathers JC Fox CR Gackowska A Oakley F Burt AD Wilson CL Anstee QM Barter MJ Masson S Elsharkawy AM Mann DA Mann J Multigenerational epigenetic adaptation of the hepatic wound-healing response. Nat Med 2012;18:1369–77 22941276 10.1038/nm.2893 PMC3489975

[22] Arai JA Feig LA Long-lasting and transgenerational effects of an environmental enrichment on memory formation. Brain Res Bull 2011;85:30–5 21078373 10.1016/j.brainresbull.2010.11.003 PMC3070197

[23] Helgason T Jonasson MR Evidence for a food additive as a cause of ketosis-prone diabetes. Lancet 1981;2:716–20 6116858 10.1016/s0140-6736(81)91048-5

[24] Leary SD Smith GD Rogers IS Reilly JJ Wells JC Ness AR Smoking during pregnancy and offspring fat and lean mass in childhood. Obesity (Silver Spring) 2006;14:2284–93 17189557 10.1038/oby.2006.268 PMC1890311

[25] Heijmans BT Tobi EW Stein AD Putter H Blauw GJ Susser ES Slagboom PE Lumey LH Persistent epigenetic differences associated with prenatal exposure to famine in humans. Proc Natl Acad Sci USA 2008;105:17046–9 18955703 10.1073/pnas.0806560105 PMC2579375

[26] Tobi EW Lumey LH Talens RP Kremer D Putter H Stein AD Slagboom PE Heijmans BT DNA methylation differences after exposure to prenatal famine are common and timing- and sex-specific. Hum Mol Genet 2009;18:4046–53 19656776 10.1093/hmg/ddp353 PMC2758137

[27] Bygren LO Kaati G Edvinsson S Longevity determined by paternal ancestors’ nutrition during their slow growth period. Acta Biotheor 2001;49:53–9 11368478 10.1023/a:1010241825519

[28] Kaati G Bygren LO Edvinsson S Cardiovascular and diabetes mortality determined by nutrition during parents’ and grandparents’ slow growth period. Eur J Hum Genet 2002;10:682–8 12404098 10.1038/sj.ejhg.5200859

[29] Pembrey ME Bygren LO Kaati G Edvinsson S Northstone K Sjostrom M Golding J Team AS Sex-specific, male-line transgenerational responses in humans. Eur J Hum Genet 2006;14:159–66 16391557 10.1038/sj.ejhg.5201538

[30] Kaati G Bygren LO Pembrey M Sjostrom M Transgenerational response to nutrition, early life circumstances and longevity. Eur J Hum Genet 2007;15:784–90 17457370 10.1038/sj.ejhg.5201832

[31] Bygren LO Tinghög P Carstensen J Edvinsson S Kaati G Pembrey ME Sjöström M Change in paternal grandmothers’ early food supply influenced cardiovascular mortality of the female grandchildren. BMC Genet 2014;15:12 24552514 10.1186/1471-2156-15-12 PMC3929550

[32] van den Berg GJ Pinger P A validation study fo transgenerational effects of childhood conditions on the third generation offspring's economic and health outcomes potentially driven by epigenetic imprinting. IZA Discussion Paper, 2014

[33] Stein AD Wang M DiGirolamo A Grajeda R Ramakrishnan U Ramirez-Zea M Yount K Martorell R Nutritional supplementation in early childhood, schooling, and intellectual functioning in adulthood: a prospective study in Guatemala. Arch Pediatr Adolesc Med 2008;162:612–8 18606931 10.1001/archpedi.162.7.612 PMC3733080

[34] Huang C Li Z Venkat Narayan KM Williamson DF Martorell R Bigger babies born to women survivors of the 1959–1961 Chinese famine: a puzzle due to survival selection? J Dev Orig Health Dis 2010;1:412–18 25142012 10.1017/S2040174410000504

[35] Lumey LH Decreased birthweights in infants after maternal in utero exposure to the Dutch famine of 1944–1945. Paediatr Perinat Epidemiol 1992;6:240–53 1584725 10.1111/j.1365-3016.1992.tb00764.x

[36] Lumey LH Stein AD Ravelli AC Timing of prenatal starvation in women and offspring birth weight: an update. Eur J Obstet Gynecol Reprod Biol 1995;63:197 8903779 10.1016/0301-2115(95)02240-6

[37] Lumey LH Stein AD Offspring birth weights after maternal intrauterine undernutrition: a comparison within sibships. Am J Epidemiol 1997;146:810–9 9384201 10.1093/oxfordjournals.aje.a009198

[38] Painter RC Osmond C Gluckman P Hanson M Phillips DI Roseboom TJ Transgenerational effects of prenatal exposure to the Dutch famine on neonatal adiposity and health in later life. BJOG 2008;115:1243–9 18715409 10.1111/j.1471-0528.2008.01822.x

[39] Northstone K Golding J Davey Smith G Miller LL Pembrey M Prepubertal start of father's smoking and increased body fat in his sons: further characterisation of paternal transgenerational responses. Eur J Hum Genet 2014 Apr 2. doi:10.1038/ejhg.2014.31. [Epub ahead of print]. 10.1038/ejhg.2014.31 PMC4085023 24690679

[40] Miller LL Pembrey M Davey Smith G Northstone K Golding J Is the growth of the fetus of a non-smoking mother influenced by the smoking of either grandmother while pregnant? PloS one 2014;9:e86781 24504157 10.1371/journal.pone.0086781 PMC3913581

[41] Misra DP Astone N Lynch CD Maternal smoking and birth weight: interaction with parity and mother's own in utero exposure to smoking. Epidemiology (Cambridge, Mass) 2005;16:288–93 10.1097/01.ede.0000158198.59544.cf 15824542

[42] Hypponen E Smith GD Power C Effects of grandmothers’ smoking in pregnancy on birth weight: intergenerational cohort study. BMJ 2003;327:898 14563745 10.1136/bmj.327.7420.898 PMC218811

[43] Li YF Langholz B Salam MT Gilliland FD Maternal and grandmaternal smoking patterns are associated with early childhood asthma. Chest 2005;127:1232–41 15821200 10.1378/chest.127.4.1232

[44] Miller LL Henderson J Northstone K Pembrey M Golding J Do grandmaternal smoking patterns influence the aetiology of childhood asthma? Chest 2014;145:1213–18 24158349 10.1378/chest.13-1371 PMC4042509

[45] Boucher BJ Ewen SW Stowers JM Betel nut (Areca catechu) consumption and the induction of glucose intolerance in adult CD1 mice and in their F1 and F2 offspring. Diabetologia 1994;37:49–55 8150230 10.1007/BF00428777

[46] Chen TH Chiu YH Boucher BJ Transgenerational effects of betel-quid chewing on the development of the metabolic syndrome in the Keelung Community-based Integrated Screening Program. Am J Clin Nutr 2006;83:688–92 16522918 10.1093/ajcn.83.3.688

[47] Drake AJ Walker BR The intergenerational effects of fetal programming: non-genomic mechanisms for the inheritance of low birth weight and cardiovascular risk. J Endocrinol 2004;180:1–16 14709139 10.1677/joe.0.1800001

[48] Anderson LM Riffle L Wilson R Travlos GS Lubomirski MS Alvord WG Preconceptional fasting of fathers alters serum glucose in offspring of mice. Nutrition 2006;22:327–31 16500559 10.1016/j.nut.2005.09.006

[49] Gabory A Roseboom TJ Moore T Moore LG Junien C Placental contribution to the origins of sexual dimorphism in health and diseases: sex chromosomes and epigenetics. Bio Sex Differences 2013;4:5 10.1186/2042-6410-4-5 PMC3618244 23514128

[50] Champagne FA Effects of stress across generations: why sex matters. Biol Psychiatry 2013;73:2–4 23217458 10.1016/j.biopsych.2012.10.004

[51] Brookes ST Whitley E Peters TJ Mulheran PA Egger M Davey Smith G Subgroup analyses in randomised controlled trials: quantifying the risks of false-positives and false-negatives. Health Technol Assess 2001;5:1–56 11701102 10.3310/hta5330

[52] Pembrey ME Male-line transgenerational responses in humans. Hum Fertil (Camb) 2010;13:268–71 21117937 10.3109/14647273.2010.524721

[53] Saavedra-Rodriguez L Feig LA Chronic social instability induces anxiety and defective social interactions across generations. Biol Psychiatry 2013;73:44–53 22906514 10.1016/j.biopsych.2012.06.035 PMC3826464

[54] Franklin TB Russig H Weiss IC Graff J Linder N Michalon A Vizi S Mansuy IM Epigenetic transmission of the impact of early stress across generations. Biol Psychiatry 2010;68:408–15 20673872 10.1016/j.biopsych.2010.05.036

[55] Gapp K Jawaid A Sarkies P Bohacek J Pelczar P Prados J Farinelli L Miska E Mansuy IM Implication of sperm RNAs in transgenerational inheritance of the effects of early trauma in mice. Nat Neurosci 2014;17:667–9 24728267 10.1038/nn.3695 PMC4333222

[56] Dunn GA Bale TL Maternal high-fat diet effects on third-generation female body size via the paternal lineage. Endocrinology 2011;152:2228–36 21447631 10.1210/en.2010-1461 PMC3100614

[57] Skinner MK Haque CG Nilsson E Bhandari R McCarrey JR Environmentally induced transgenerational epigenetic reprogramming of primordial germ cells and the subsequent germ line. PloS One 2013;8:e66318 23869203 10.1371/journal.pone.0066318 PMC3712023

[58] Cheng CY Mruk DD Cell junction dynamics in the testis: Sertoli-germ cell interactions and male contraceptive development. Physiol Rev 2002;82: 825–74 12270945 10.1152/physrev.00009.2002

[59] Bromfield JJ Schjenken JE Chin PY Care AS Jasper MJ Robertson SA Maternal tract factors contribute to paternal seminal fluid impact on metabolic phenotype in offspring. Proc Natl Acad Sci USA 2014;111:2200–5 24469827 10.1073/pnas.1305609111 PMC3926084

[60] Novakovic B Saffery R The importance of the intrauterine environment in shaping the human neonatal epigenome. Epigenomics 2013;5:1–4 23414310 10.2217/epi.12.77

[61] Alcazar RM Lin R Fire AZ Transmission dynamics of heritable silencing induced by double-stranded RNA in Caenorhabditis elegans. Genetics 2008;180: 1275–88 18757930 10.1534/genetics.108.089433 PMC2581934

[62] Reik W Walter J Genomic imprinting: parental influence on the genome. Nat Rev 2001;2:21–32 10.1038/35047554 11253064

[63] Seisenberger S Peat JR Hore TA Santos F Dean W Reik W Reprogramming DNA methylation in the mammalian life cycle: building and breaking epigenetic barriers. Philos Trans R Soc Lond B Biol Sci 2013;368:20110330 23166394 10.1098/rstb.2011.0330 PMC3539359

[64] Steegers-Theunissen RP Obermann-Borst SA Kremer D Lindemans J Siebel C Steegers EA Slagboom PE Heijmans BT Periconceptional maternal folic acid use of 400 microg per day is related to increased methylation of the IGF2 gene in the very young child. PloS One 2009;4:e7845 19924280 10.1371/journal.pone.0007845 PMC2773848

[65] Haycock PC Ramsay M Exposure of mouse embryos to ethanol during preimplantation development: effect on DNA methylation in the H19 imprinting control region. Biol Reprod 2009;81:618–27 19279321 10.1095/biolreprod.108.074682

[66] Stouder C Deutsch S Paoloni-Giacobino A Superovulation in mice alters the methylation pattern of imprinted genes in the sperm of the offspring. Reprod Toxicol 2009;28:536–41 19549566 10.1016/j.reprotox.2009.06.009

[67] Ollikainen M Smith KR Joo EJ Ng HK Andronikos R Novakovic B Abdul Aziz NK Carlin JB Morley R Saffery R Craig JM DNA methylation analysis of multiple tissues from newborn twins reveals both genetic and intrauterine components to variation in the human neonatal epigenome. Hum Mol Genet 2010;19:4176–88 20699328 10.1093/hmg/ddq336

[68] Ba Y Yu H Liu F Geng X Zhu C Zhu Q Zheng T Ma S Wang G Li Z Zhang Y Relationship of folate, vitamin B12 and methylation of insulin-like growth factor-II in maternal and cord blood. Eur J Clin Nutr 2011;65:480–5 21245875 10.1038/ejcn.2010.294 PMC3071883

[69] Hoyo C Murtha AP Schildkraut JM Jirtle RL Demark-Wahnefried W Forman MR Iversen ES Kurtzberg J Overcash F Huang Z Murphy SK Methylation variation at IGF2 differentially methylated regions and maternal folic acid use before and during pregnancy. Epigenetics 2011;6:928–36 21636975 10.4161/epi.6.7.16263 PMC3154433

[70] Murphy SK Huang Z Hoyo C Differentially methylated regions of imprinted genes in prenatal, perinatal and postnatal human tissues. PloS One 2012;7:e40924 22808284 10.1371/journal.pone.0040924 PMC3396645

[71] Cooper WN Khulan B Owens S Elks CE Seidel V Prentice AM Belteki G Ong KK Affara NA Constancia M Dunger DB DNA methylation profiling at imprinted loci after periconceptional micronutrient supplementation in humans: results of a pilot randomized controlled trial. FASEB J 2012;26:1782–90 22267336 10.1096/fj.11-192708

[72] Loke YJ Galati JC Morley R Joo JE Novakovic B Li X Weinrich B Carson N Ollikainen M Ng HK Andronikos R Aziz NK Saffery R Craig JM Association of maternal environmental and nutrient supply line factors with DNA methylation at the imprinted IGF2/H19 locus in multiple tissues of newborn twins. Epigenetics 2013;8:1069–79 23917818 10.4161/epi.25908 PMC3891688

[73] Hackett JA Sengupta R Zylicz JJ Murakami K Lee C Down TA Surani MA Germline DNA demethylation dynamics and imprint erasure through 5-hydroxymethylcytosine. Science (New York, NY) 2013;339:448–52 10.1126/science.1229277 PMC3847602 23223451

[74] Mukamel Z Tanay A Hypomethylation marks enhancers within transposable elements. Nat Genet 2013;45:717–8 23800863 10.1038/ng.2680

[75] Smallwood SA Tomizawa S Krueger F Ruf N Carli N Segonds-Pichon A Sato S Hata K Andrews SR Kelsey G Dynamic CpG island methylation landscape in oocytes and preimplantation embryos. Nat Genet 2011;43:811–4 21706000 10.1038/ng.864 PMC3146050

[76] Tomizawa S Nowacka-Woszuk J Kelsey G DNA methylation establishment during oocyte growth: mechanisms and significance. Int J Dev Biol 2012;56:867–75 23417409 10.1387/ijdb.120152gk

[77] Hammoud SS Nix DA Zhang H Purwar J Carrell DT Cairns BR Distinctive chromatin in human sperm packages genes for embryo development. Nature 2009;460:473–8 19525931 10.1038/nature08162 PMC2858064

[78] Brykczynska U Hisano M Erkek S Ramos L Oakeley EJ Roloff TC Beisel C Schubeler D Stadler MB Peters AH Repressive and active histone methylation mark distinct promoters in human and mouse spermatozoa. Nat Struct Mol Biol 2010;17:679–87 20473313 10.1038/nsmb.1821

[79] Carrell DT Hammoud SS The human sperm epigenome and its potential role in embryonic development. Mol Hum Reprod 2010;16:37–47 19906823 10.1093/molehr/gap090

[80] Gannon JR Emery BR Jenkins TG Carrell DT The sperm epigenome: implications for the embryo. Adv Exp Med Biol 2014;791:53–66 23955672 10.1007/978-1-4614-7783-9_4

[81] Wei Y Yang CR Wei YP Zhao ZA Hou Y Schatten H Sun QY Paternally induced transgenerational inheritance of susceptibility to diabetes in mammals. Proc Natl Acad Sci USA 2014 10.1073/pnas.1321195111 PMC3918818 24449870

[82] Cortez D Marin R Toledo-Flores D Froidevaux L Liechti A Waters PD Grutzner F Kaessmann H Origins and functional evolution of Y chromosomes across mammals. Nature 2014;508:488–93 24759410 10.1038/nature13151

[83] Sendler E Johnson GD Mao S Goodrich RJ Diamond MP Hauser R Krawetz SA Stability, delivery and functions of human sperm RNAs at fertilization. Nucleic Acids Res 2013;41:4104–17 23471003 10.1093/nar/gkt132 PMC3627604

[84] Fullston T Ohlsson Teague EM Palmer NO DeBlasio MJ Mitchell M Corbett M Print CG Owens JA Lane M Paternal obesity initiates metabolic disturbances in two generations of mice with incomplete penetrance to the F2 generation and alters the transcriptional profile of testis and sperm microRNA content. FASEB J 2013;27:4226–43 23845863 10.1096/fj.12-224048

[85] Rodgers AB Morgan CP Bronson SL Revello S Bale TL Paternal stress exposure alters sperm microRNA content and reprograms offspring HPA stress axis regulation. J Neurosci 2013;33:9003–12 23699511 10.1523/JNEUROSCI.0914-13.2013 PMC3712504

[86] Marczylo EL Amoako AA Konje JC Gant TW Marczylo TH Smoking induces differential miRNA expression in human spermatozoa: a potential transgenerational epigenetic concern? Epigenetics 2012;7:432–9 22441141 10.4161/epi.19794

[87] Gertz J Varley KE Reddy TE Bowling KM Pauli F Parker SL Kucera KS Willard HF Myers RM Analysis of DNA methylation in a three-generation family reveals widespread genetic influence on epigenetic regulation. PLoS Genetics 2011;7:e1002228 21852959 10.1371/journal.pgen.1002228 PMC3154961

[88] Hitchins MP Wong JJ Suthers G Suter CM Martin DI Hawkins NJ Ward RL Inheritance of a cancer-associated MLH1 germ-line epimutation. N Engl J Med 2007;356:697–705 17301300 10.1056/NEJMoa064522

[89] Hitchins MP Rapkins RW Kwok CT Srivastava S Wong JJ Khachigian LM Polly P Goldblatt J Ward RL Dominantly inherited constitutional epigenetic silencing of MLH1 in a cancer-affected family is linked to a single nucleotide variant within the 5′UTR. Cancer Cell 2011;20:200–13 21840485 10.1016/j.ccr.2011.07.003

[90] Kwok CT Vogelaar IP van Zelst-Stams WA Mensenkamp AR Ligtenberg MJ Rapkins RW Ward RL Chun N Ford JM Ladabaum U McKinnon WC Greenblatt MS Hitchins MP The MLH1 c.-27C>A and c.85G>T variants are linked to dominantly inherited MLH1 epimutation and are borne on a European ancestral haplotype. Eur J Hum Genet 2014;22:617–24 24084575 10.1038/ejhg.2013.200 PMC3992563

[91] Teh AL Pan H Chen L Ong ML Dogra S Wong J Macisaac JL Mah SM McEwen LM Saw SM Godfrey KM Chong YS Kwek K Kwoh CK Soh SE Chong MF Barton S Karnani N Cheong CY Buschdorf JP Stunkel W Kobor MS Meaney MJ Gluckman PD Holbrook JD The effect of genotype and in utero environment on inter-individual variation in neonate DNA methylomes. Genome Research 2014;24:1064–74 24709820 10.1101/gr.171439.113 PMC4079963

[92] Yazbek SN Spiezio SH Nadeau JH Buchner DA Ancestral paternal genotype controls body weight and food intake for multiple generations. Hum Mol Genet 2010;19:4134–44 20696673 10.1093/hmg/ddq332 PMC2951864

[93] Hara MR Kovacs JJ Whalen EJ Rajagopal S Strachan RT Grant W Towers AJ Williams B Lam CM Xiao K Shenoy SK Gregory SG Ahn S Duckett DR Lefkowitz RJ A stress response pathway regulates DNA damage through beta2-adrenoreceptors and beta-arrestin-1. Nature 2011;477:349–53 21857681 10.1038/nature10368 PMC3628753

[94] Padmanabhan N Jia D Geary-Joo C Wu X Ferguson-Smith AC Fung E Bieda MC Snyder FF Gravel RA Cross JC Watson ED Mutation in folate metabolism causes epigenetic instability and transgenerational effects on development. Cell 2013;155:81–93 24074862 10.1016/j.cell.2013.09.002 PMC3844871

[95] Nordenfelt L Causation: an essay. Stockholm: Akademilitteratur, 1981

[96] Lindahl BIB On causal attribution. Stockholm: Stockholm University (Acta Universitatis Stockholmiensis), 2009

[97] Beyan H Down T Ramagopalan S Uvebrant K Nilsson A Holland M Gemma C Giovannoni G Boehm B Ebers G Lernmark A Cillio C Leslie D Rakyan V Guthrie card methylomics identifies temporally stable epialleles that are present at birth in humans. Genome Res 2012;22:2138–45 22919074 10.1101/gr.134304.111 PMC3483543

[98] Grasedieck S Sorrentino A Langer C Buske C Dohner H Mertens D Kuchenbauer F Circulating microRNAs in hematological diseases: principles, challenges, and perspectives. Blood 2013;121:4977–84 23550041 10.1182/blood-2013-01-480079

[99] Bygren LO Kaati G Edvinsson S Pembrey ME Reply to senn. Eur J Hum Genet 2006;14:1149–5016823395

[100] Senn S Epigenetics or ephemeral genetics? Eur J Hum Genet 2006;14:1149; author reply 49–50 16823395 10.1038/sj.ejhg.5201683

[101] Bygren LO Edvinsson S Brostrom G Change in food availability during pregnancy: Is it related to adult sudden death from cerebro- and cardiovascular disease in offspring? Am J Hum Biol 2000;12:447–53 11534035 10.1002/1520-6300(200007/08)12:4<447::AID-AJHB3>3.0.CO;2-M

[102] Wisborg K Kesmodel U Henriksen TB Olsen SF Secher NJ Exposure to tobacco smoke in utero and the risk of stillbirth and death in the first year of life. Am J Epidemiol 2001;154:322–7 11495855 10.1093/aje/154.4.322

[103] Boyd A Golding J Macleod J Lawlor DA Fraser A Henderson J Molloy L Ness A Ring S Davey Smith G Cohort profile: the ‘children of the 90s’—the index offspring of the Avon longitudinal study of parents and children. Int J Epidemiol 2013;42:111–27 22507743 10.1093/ije/dys064 PMC3600618

[104] Elliott J Shepherd P Cohort profile: 1970 British Birth Cohort (BCS70). Int J Epidemiol 2006;35:836–43 16931528 10.1093/ije/dyl174

[105] Wright J Small N Raynor P Tuffnell D Bhopal R Cameron N Fairley L Lawlor DA Parslow R Petherick ES Pickett KE Waiblinger D West J, Born in Bradford Scientific Collaborators G. Cohort profile: the born in Bradford multi-ethnic family cohort study. Int J Epidemiol 2013;42:978–91 23064411 10.1093/ije/dys112

[106] Olsen J Sorensen HT The Danish national birth cohort—a valuable tool for pharmacoepidemiology in pregnancy. Int J Risk Saf Med 1997;10:197–8 23511373 10.3233/JRS-1997-10307

[107] Connelly R Platt L Cohort profile: UK millennium Cohort study (MCS). Int J Epidemiol 2014 Feb 17. [Epub ahead of print] PubMed PMID: 24550246. 10.1093/ije/dyu001 24550246

[108] Magnus P Irgens LM Haug K Nystad W Skjaerven R Stoltenberg C, MoBa Study G. Cohort profile: the Norwegian mother and child Cohort study (MoBa). Int J Epidemiol 2006;35:1146–50 16926217 10.1093/ije/dyl170

[109] Peckham CS A national study of child development (NCDS 1958 cohort). Preliminary findings in a national sample of 11-year-old children. Proc R Soc Med 1973;66:701–3 4741414 10.1177/003591577306600749 PMC1645076

[110] Heshmati A Mishra G Koupil I Childhood and adulthood socio-economic position and hypertensive disorders in pregnancy: the Uppsala Birth Cohort Multigenerational Study. J Epidemiol Community Health 2013;67:939–46 23729327 10.1136/jech-2012-202149

